# 2-Eth­oxy-6-{[1-(3-eth­oxy-2-hy­droxy­benz­yl)-1*H*-benzimidazol-2-yl]meth­yl}phenol nitro­methane monosolvate

**DOI:** 10.1107/S1600536812023665

**Published:** 2012-05-31

**Authors:** Kwang Ha

**Affiliations:** a School of Applied Chemical Engineering, The Research Institute of Catalysis, Chonnam National University, Gwangju 500-757, Republic of Korea

## Abstract

In the title solvate, C_24_H_24_N_2_O_4_·CH_3_NO_2_, the benzene ring of the 2-eth­oxy-6-methyl­phenol substituent is approximately perpendicular to the nearly planar benzimidazole ring [maximum deviation = 0.021 (2) Å], making a dihedral angle of 84.32 (7)°. The benzene ring of the 2-eth­oxy­phenol group is somewhat inclined to the benzimidazole ring plane by 28.03 (5)°. The dihedral angle between the benzene rings is 82.20 (9)°. The compound reveals strong intra­molecular O—H⋯N and O—H⋯O hydrogen bonds, forming six- and five-membered rings, respectively. In the crystal, mol­ecules are connected by bifurcated O—H⋯(O,O) hydrogen bonds, forming chains along the *b* axis.

## Related literature

For the crystal structure of the meth­oxy derivative of the title compound, see: Al-Douh *et al.* (2009 ▶). For the crystal structure of the title compound as an acetonitrile monosolvate, see: Ha (2012 ▶).
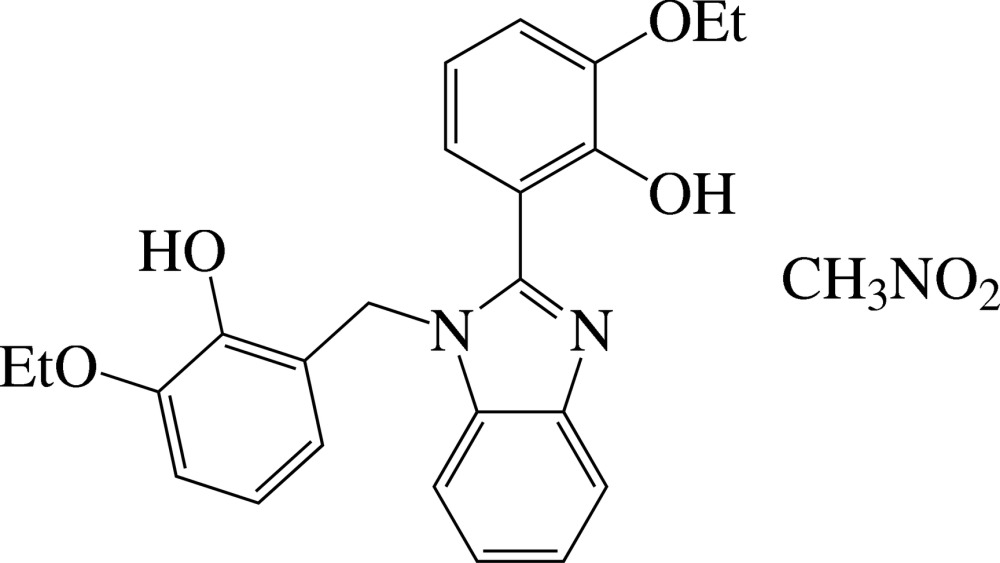


## Experimental

### 

#### Crystal data

C_24_H_24_N_2_O_4_·CH_3_NO_2_
*M* *_r_* = 465.50
Monoclinic, 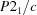

*a* = 7.5151 (7) Å
*b* = 19.6463 (17) Å
*c* = 16.2578 (15) Å
β = 99.898 (2)°
*V* = 2364.6 (4) Å^3^
*Z* = 4
Mo *K*α radiation
μ = 0.09 mm^−1^
*T* = 273 K
0.36 × 0.20 × 0.13 mm

#### Data collection

Bruker SMART 1000 CCD diffractometer
Absorption correction: multi-scan (*SADABS*; Bruker, 2000 ▶) *T*_min_ = 0.856, *T*_max_ = 1.000
17462 measured reflections
5846 independent reflections
2523 reflections with *I* > 2σ(*I*)
*R*_int_ = 0.090

#### Refinement

*R*[*F* ^2^ > 2σ(*F* ^2^)] = 0.064
*wR*(*F* ^2^) = 0.157
*S* = 0.95
5846 reflections
318 parameters
H atoms treated by a mixture of independent and constrained refinement
Δρ_max_ = 0.29 e Å^−3^
Δρ_min_ = −0.22 e Å^−3^

### 

Data collection: *SMART* (Bruker, 2000 ▶); cell refinement: *SAINT* (Bruker, 2000 ▶); data reduction: *SAINT*; program(s) used to solve structure: *SHELXS97* (Sheldrick, 2008 ▶); program(s) used to refine structure: *SHELXL97* (Sheldrick, 2008 ▶); molecular graphics: *ORTEP-3* (Farrugia, 1997 ▶) and *PLATON* (Spek, 2009 ▶); software used to prepare material for publication: *SHELXL97*.

## Supplementary Material

Crystal structure: contains datablock(s) global, I. DOI: 10.1107/S1600536812023665/tk5103sup1.cif

Structure factors: contains datablock(s) I. DOI: 10.1107/S1600536812023665/tk5103Isup2.hkl

Supplementary material file. DOI: 10.1107/S1600536812023665/tk5103Isup3.cml

Additional supplementary materials:  crystallographic information; 3D view; checkCIF report

## Figures and Tables

**Table 1:** Hydrogen-bond geometry (Å, °)

*D*—H⋯*A*	*D*—H	H⋯*A*	*D*⋯*A*	*D*—H⋯*A*
O1—H1*O*⋯N1	0.92 (4)	1.75 (4)	2.596 (3)	152 (3)
O3—H3*O*⋯O4	0.99 (3)	2.27 (3)	2.710 (2)	106 (2)
O3—H3*O*⋯O1^i^	0.99 (3)	1.91 (3)	2.819 (2)	151 (2)
O3—H3*O*⋯O2^i^	0.99 (3)	2.36 (3)	3.012 (3)	122 (2)

Symmetry code: (i) 
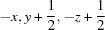
.

**Fig. 1.**
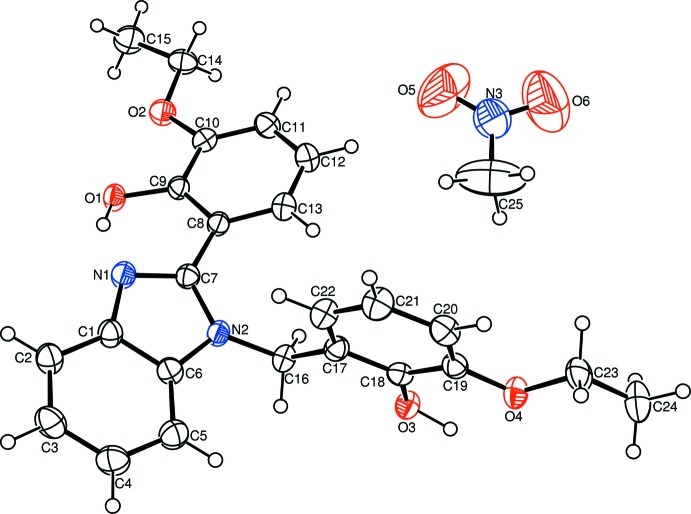
A structure detail of the title compound, with atom numbering. Displacement ellipsoids are drawn at the 40% probability level for non-H atoms.

**Fig. 2.**
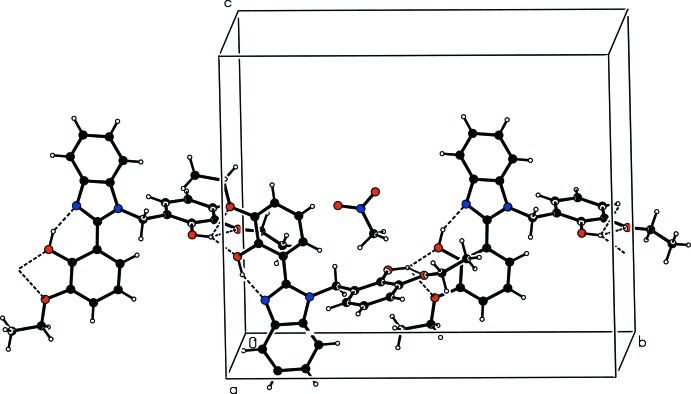
A partial view along the a axis of the crystal packing of the title compound. Intra- and intermolecular O—H···N and O—H···O hydrogen-bonds are shown as dashed lines.

## supplementary crystallographic information

### Comment

The crystal structures of the related compound, 2-[1-(2-hydroxy-3-methoxybenzyl)-1*H*-benzimidazol-2-yl]-6-methoxyphenol monohydrate, and the title compound as an acetonitrile monosolvate have been reported previously (Al-Douh *et al.*, 2009; Ha, 2012).

The title compound, C_24_H_24_N_2_O_4_.CH_3_NO_2_, contains a disubstituted benzimidazole molecule and a lattice solvent molecule (Fig. 1). The benzene ring (C17–C22) of the 2-ethoxy-6-methylphenol substituent is approximately perpendicular to the nearly planar benzimidazole ring system [maximum deviation = 0.021 (2) Å], making a dihedral angle of 84.32 (7)°. The benzene ring (C8–C13) of the 2-ethoxyphenol group is somewhat inclined to the benzimidazole ring plane by 28.03 (5)°. The dihedral angle between the benzene rings is 82.20 (9)°. The compound reveals strong intramolecular O—H···N and O—H···O hydrogen bonds, forming six- and five-membered rings, respectively (Fig. 2 and Table 1). In the crystal, molecules are connected by bifurcated O—H···(O,O) hydrogen bonds, forming chains along the *b* axis (Fig. 2 and Table 1).

### Experimental

1,2-Phenylenediamine (0.7568 g, 6.998 mmol) and 3-ethoxysalicylaldehyde (2.3269 g, 14.003 mmol) in EtOH (20 ml) were stirred for 5 h at room temperature. After evaporation of the solvent, the residue was recrystallized from a mixture of acetone and ether at 188 K, to give an orange powder (1.9139 g). Crystals suitable for X-ray analysis were obtained by slow evaporation from its CH_3_NO_2_ solution at room temperature.

### Refinement

Carbon-bound H atoms were positioned geometrically and allowed to ride on their respective parent atoms: C—H = 0.93, 0.96 and 0.97 Å for CH, CH_3_ and CH_2_ groups, respectively, with *U*_iso_(H) = 1.2*U*_eq_(C) or 1.5*U*_eq_(methyl C). The hydroxy-H atoms were located from a difference Fourier map and refined freely. A number of reflections, (0 1 1), (0 3 2), (5 12 14), (2 11 15), (1 6 19), (2 13 16), (1 4 0) and (3 22 1), were omitted from the final refinement owing to poor agreement.

### Crystal data

**Table:**

C_24_H_24_N_2_O_4_·CH_3_NO_2_	*F*(000) = 984
*M**_r_* = 465.50	*D*_x_ = 1.308 Mg m^−^^3^
Monoclinic, *P*2_1_/*c*	Mo *K*α radiation, λ = 0.71073 Å
Hall symbol: -P 2ybc	Cell parameters from 2443 reflections
*a* = 7.5151 (7) Å	θ = 2.8–22.7°
*b* = 19.6463 (17) Å	µ = 0.09 mm^−^^1^
*c* = 16.2578 (15) Å	*T* = 273 K
β = 99.898 (2)°	Block, orange
*V* = 2364.6 (4) Å^3^	0.36 × 0.20 × 0.13 mm
*Z* = 4	

### Data collection

**Table:**

Bruker SMART 1000 CCD diffractometer	5846 independent reflections
Radiation source: fine-focus sealed tube	2523 reflections with *I* > 2σ(*I*)
Graphite monochromator	*R*_int_ = 0.090
φ and ω scans	θ_max_ = 28.3°, θ_min_ = 2.1°
Absorption correction: multi-scan (*SADABS*; Bruker, 2000)	*h* = −9→10
*T*_min_ = 0.856, *T*_max_ = 1.000	*k* = −26→23
17462 measured reflections	*l* = −21→21

### Refinement

**Table:**

Refinement on *F*^2^	Primary atom site location: structure-invariant direct methods
Least-squares matrix: full	Secondary atom site location: difference Fourier map
*R*[*F*^2^ > 2σ(*F*^2^)] = 0.064	Hydrogen site location: inferred from neighbouring sites
*wR*(*F*^2^) = 0.157	H atoms treated by a mixture of independent and constrained refinement
*S* = 0.95	*w* = 1/[σ^2^(*F*_o_^2^) + (0.0503*P*)^2^] where *P* = (*F*_o_^2^ + 2*F*_c_^2^)/3
5846 reflections	(Δ/σ)_max_ < 0.001
318 parameters	Δρ_max_ = 0.29 e Å^−^^3^
0 restraints	Δρ_min_ = −0.22 e Å^−^^3^

### Special details

**Table:**

Geometry. All e.s.d.'s (except the e.s.d. in the dihedral angle between two l.s. planes) are estimated using the full covariance matrix. The cell e.s.d.'s are taken into account individually in the estimation of e.s.d.'s in distances, angles and torsion angles; correlations between e.s.d.'s in cell parameters are only used when they are defined by crystal symmetry. An approximate (isotropic) treatment of cell e.s.d.'s is used for estimating e.s.d.'s involving l.s. planes.
Refinement. Refinement of *F*^2^ against ALL reflections. The weighted *R*-factor *wR* and goodness of fit *S* are based on *F*^2^, conventional *R*-factors *R* are based on *F*, with *F* set to zero for negative *F*^2^. The threshold expression of *F*^2^ > σ(*F*^2^) is used only for calculating *R*-factors(gt) *etc*. and is not relevant to the choice of reflections for refinement. *R*-factors based on *F*^2^ are statistically about twice as large as those based on *F*, and *R*- factors based on ALL data will be even larger.

### Fractional atomic coordinates and isotropic or equivalent isotropic displacement parameters (Å^2^)

**Table:**

	*x*	*y*	*z*	*U*_iso_*/*U*_eq_	
O1	0.1517 (3)	−0.00824 (8)	0.25918 (12)	0.0364 (5)	
H1O	0.166 (5)	0.0034 (17)	0.206 (2)	0.105 (14)*	
O2	0.1909 (3)	−0.02200 (9)	0.41939 (11)	0.0421 (5)	
O3	−0.0023 (2)	0.36913 (9)	0.19378 (11)	0.0367 (5)	
H3O	−0.032 (4)	0.4180 (15)	0.1965 (19)	0.076 (10)*	
O4	0.2353 (2)	0.47405 (8)	0.21047 (12)	0.0399 (5)	
N1	0.2058 (3)	0.06287 (10)	0.13119 (14)	0.0362 (6)	
N2	0.1347 (3)	0.17347 (10)	0.13095 (13)	0.0329 (6)	
C1	0.1707 (4)	0.08554 (13)	0.04930 (17)	0.0352 (7)	
C2	0.1729 (4)	0.05148 (14)	−0.02530 (18)	0.0424 (8)	
H2	0.2012	0.0054	−0.0258	0.051*	
C3	0.1320 (4)	0.08766 (15)	−0.09819 (18)	0.0475 (8)	
H3	0.1329	0.0657	−0.1488	0.057*	
C4	0.0889 (4)	0.15692 (15)	−0.09826 (19)	0.0494 (8)	
H4	0.0629	0.1801	−0.1488	0.059*	
C5	0.0841 (4)	0.19139 (14)	−0.02510 (19)	0.0453 (8)	
H5	0.0545	0.2373	−0.0249	0.054*	
C6	0.1256 (4)	0.15445 (13)	0.04832 (17)	0.0345 (7)	
C7	0.1872 (3)	0.11683 (12)	0.17827 (16)	0.0312 (6)	
C8	0.2174 (3)	0.11250 (12)	0.26941 (16)	0.0311 (6)	
C9	0.1955 (3)	0.04905 (12)	0.30544 (16)	0.0298 (6)	
C10	0.2224 (4)	0.04219 (13)	0.39271 (17)	0.0336 (7)	
C11	0.2786 (4)	0.09698 (14)	0.44263 (18)	0.0403 (7)	
H11	0.2974	0.0924	0.5004	0.048*	
C12	0.3074 (4)	0.15947 (14)	0.40681 (18)	0.0433 (8)	
H12	0.3477	0.1963	0.4409	0.052*	
C13	0.2772 (4)	0.16734 (13)	0.32187 (18)	0.0396 (7)	
H13	0.2965	0.2095	0.2989	0.048*	
C14	0.2320 (4)	−0.03579 (14)	0.50701 (17)	0.0444 (8)	
H14A	0.1562	−0.0085	0.5368	0.053*	
H14B	0.3574	−0.0251	0.5285	0.053*	
C15	0.1970 (4)	−0.11004 (14)	0.51828 (18)	0.0537 (9)	
H15A	0.0730	−0.1201	0.4961	0.080*	
H15B	0.2216	−0.1211	0.5766	0.080*	
H15C	0.2741	−0.1365	0.4893	0.080*	
C16	0.0750 (4)	0.23959 (12)	0.15603 (17)	0.0355 (7)	
H16A	0.0374	0.2351	0.2099	0.043*	
H16B	−0.0293	0.2539	0.1161	0.043*	
C17	0.2187 (4)	0.29402 (12)	0.16189 (16)	0.0323 (7)	
C18	0.1706 (4)	0.35914 (12)	0.18154 (16)	0.0313 (6)	
C19	0.2982 (4)	0.41196 (13)	0.18991 (16)	0.0335 (6)	
C20	0.4725 (4)	0.39823 (14)	0.17722 (17)	0.0408 (7)	
H20	0.5579	0.4329	0.1821	0.049*	
C21	0.5192 (4)	0.33281 (14)	0.15726 (18)	0.0455 (8)	
H21	0.6363	0.3236	0.1491	0.055*	
C22	0.3929 (4)	0.28132 (13)	0.14944 (18)	0.0414 (7)	
H22	0.4252	0.2376	0.1357	0.050*	
C23	0.3662 (4)	0.52643 (13)	0.2358 (2)	0.0467 (8)	
H23A	0.4226	0.5401	0.1890	0.056*	
H23B	0.4595	0.5098	0.2799	0.056*	
C24	0.2705 (4)	0.58586 (14)	0.2663 (2)	0.0598 (10)	
H24A	0.1764	0.6012	0.2226	0.090*	
H24B	0.3550	0.6222	0.2820	0.090*	
H24C	0.2187	0.5723	0.3138	0.090*	
O5	0.6407 (5)	0.27813 (18)	0.4851 (3)	0.1619 (18)	
O6	0.6611 (6)	0.3759 (2)	0.5301 (2)	0.1664 (18)	
N3	0.6350 (4)	0.3364 (2)	0.4748 (3)	0.0808 (10)	
C25	0.6008 (6)	0.3659 (3)	0.3918 (3)	0.133 (2)	
H25A	0.4821	0.3856	0.3815	0.200*	
H25B	0.6087	0.3311	0.3511	0.200*	
H25C	0.6889	0.4006	0.3877	0.200*	

### Atomic displacement parameters (Å^2^)

**Table:**

	*U*^11^	*U*^22^	*U*^33^	*U*^12^	*U*^13^	*U*^23^
O1	0.0477 (13)	0.0267 (10)	0.0354 (12)	−0.0027 (8)	0.0084 (10)	−0.0019 (9)
O2	0.0549 (14)	0.0404 (11)	0.0313 (11)	0.0011 (9)	0.0082 (10)	0.0044 (9)
O3	0.0338 (12)	0.0312 (11)	0.0459 (12)	0.0002 (8)	0.0094 (10)	−0.0025 (9)
O4	0.0400 (13)	0.0298 (10)	0.0509 (13)	−0.0062 (9)	0.0106 (10)	−0.0049 (9)
N1	0.0440 (16)	0.0308 (12)	0.0345 (14)	0.0011 (10)	0.0083 (12)	−0.0006 (11)
N2	0.0409 (15)	0.0252 (12)	0.0327 (14)	0.0009 (10)	0.0070 (11)	−0.0007 (10)
C1	0.0424 (18)	0.0322 (15)	0.0321 (16)	−0.0024 (13)	0.0100 (14)	−0.0017 (13)
C2	0.050 (2)	0.0393 (16)	0.0385 (19)	−0.0033 (14)	0.0103 (15)	−0.0039 (14)
C3	0.056 (2)	0.056 (2)	0.0324 (18)	−0.0049 (16)	0.0112 (16)	−0.0025 (15)
C4	0.062 (2)	0.051 (2)	0.0354 (19)	−0.0020 (16)	0.0086 (16)	0.0086 (16)
C5	0.056 (2)	0.0380 (17)	0.042 (2)	0.0000 (14)	0.0088 (16)	0.0043 (15)
C6	0.0391 (18)	0.0341 (16)	0.0310 (16)	−0.0049 (12)	0.0076 (13)	0.0013 (13)
C7	0.0301 (16)	0.0298 (14)	0.0338 (16)	−0.0002 (12)	0.0055 (13)	−0.0008 (13)
C8	0.0299 (16)	0.0268 (14)	0.0360 (16)	0.0012 (11)	0.0045 (13)	−0.0028 (12)
C9	0.0280 (16)	0.0285 (14)	0.0326 (16)	0.0019 (11)	0.0047 (12)	−0.0041 (12)
C10	0.0323 (17)	0.0324 (15)	0.0366 (18)	0.0021 (12)	0.0070 (13)	0.0027 (13)
C11	0.0438 (19)	0.0438 (17)	0.0326 (16)	0.0002 (14)	0.0044 (14)	−0.0019 (14)
C12	0.048 (2)	0.0406 (17)	0.0402 (19)	−0.0014 (14)	0.0052 (15)	−0.0089 (14)
C13	0.0437 (19)	0.0324 (16)	0.0424 (19)	−0.0016 (13)	0.0065 (15)	−0.0027 (13)
C14	0.047 (2)	0.0526 (19)	0.0332 (18)	0.0012 (14)	0.0060 (15)	0.0065 (14)
C15	0.067 (2)	0.053 (2)	0.0408 (19)	0.0023 (16)	0.0111 (17)	0.0124 (15)
C16	0.0391 (18)	0.0275 (14)	0.0405 (17)	0.0010 (12)	0.0088 (14)	−0.0017 (12)
C17	0.0357 (18)	0.0303 (14)	0.0314 (16)	0.0014 (12)	0.0071 (13)	0.0018 (12)
C18	0.0308 (17)	0.0352 (15)	0.0271 (15)	−0.0006 (12)	0.0028 (12)	0.0027 (12)
C19	0.0369 (18)	0.0338 (15)	0.0291 (16)	−0.0024 (13)	0.0041 (13)	0.0001 (12)
C20	0.0394 (19)	0.0452 (17)	0.0384 (17)	−0.0081 (14)	0.0080 (14)	0.0010 (14)
C21	0.0402 (19)	0.0466 (18)	0.052 (2)	0.0019 (15)	0.0148 (16)	−0.0008 (15)
C22	0.043 (2)	0.0357 (16)	0.0463 (19)	0.0018 (14)	0.0101 (15)	−0.0004 (13)
C23	0.042 (2)	0.0415 (17)	0.055 (2)	−0.0142 (14)	0.0040 (16)	−0.0075 (15)
C24	0.059 (2)	0.0447 (19)	0.077 (3)	−0.0144 (16)	0.016 (2)	−0.0212 (17)
O5	0.123 (3)	0.078 (2)	0.279 (5)	−0.026 (2)	0.018 (3)	0.051 (3)
O6	0.212 (5)	0.166 (4)	0.118 (3)	−0.030 (3)	0.021 (3)	−0.050 (3)
N3	0.067 (2)	0.074 (3)	0.098 (3)	−0.0158 (19)	0.006 (2)	0.000 (2)
C25	0.085 (4)	0.232 (6)	0.083 (4)	0.034 (4)	0.017 (3)	0.060 (4)

### Geometric parameters (Å, º)

**Table:**

O1—C9	1.362 (3)	C13—H13	0.9300
O1—H1O	0.92 (4)	C14—C15	1.499 (4)
O2—C10	1.367 (3)	C14—H14A	0.9700
O2—C14	1.431 (3)	C14—H14B	0.9700
O3—C18	1.362 (3)	C15—H15A	0.9600
O3—H3O	0.99 (3)	C15—H15B	0.9600
O4—C19	1.370 (3)	C15—H15C	0.9600
O4—C23	1.434 (3)	C16—C17	1.511 (3)
N1—C7	1.329 (3)	C16—H16A	0.9700
N1—C1	1.386 (3)	C16—H16B	0.9700
N2—C7	1.372 (3)	C17—C22	1.381 (4)
N2—C6	1.385 (3)	C17—C18	1.382 (3)
N2—C16	1.455 (3)	C18—C19	1.403 (3)
C1—C2	1.388 (4)	C19—C20	1.387 (4)
C1—C6	1.395 (3)	C20—C21	1.385 (3)
C2—C3	1.371 (4)	C20—H20	0.9300
C2—H2	0.9300	C21—C22	1.378 (4)
C3—C4	1.399 (4)	C21—H21	0.9300
C3—H3	0.9300	C22—H22	0.9300
C4—C5	1.374 (4)	C23—C24	1.500 (4)
C4—H4	0.9300	C23—H23A	0.9700
C5—C6	1.386 (4)	C23—H23B	0.9700
C5—H5	0.9300	C24—H24A	0.9600
C7—C8	1.462 (4)	C24—H24B	0.9600
C8—C9	1.399 (3)	C24—H24C	0.9600
C8—C13	1.399 (3)	O5—N3	1.157 (4)
C9—C10	1.405 (3)	O6—N3	1.178 (4)
C10—C11	1.370 (3)	N3—C25	1.450 (5)
C11—C12	1.392 (3)	C25—H25A	0.9600
C11—H11	0.9300	C25—H25B	0.9600
C12—C13	1.369 (4)	C25—H25C	0.9600
C12—H12	0.9300		
			
C9—O1—H1O	105 (2)	H14A—C14—H14B	108.6
C10—O2—C14	118.1 (2)	C14—C15—H15A	109.5
C18—O3—H3O	112.0 (18)	C14—C15—H15B	109.5
C19—O4—C23	117.4 (2)	H15A—C15—H15B	109.5
C7—N1—C1	106.0 (2)	C14—C15—H15C	109.5
C7—N2—C6	106.7 (2)	H15A—C15—H15C	109.5
C7—N2—C16	129.8 (2)	H15B—C15—H15C	109.5
C6—N2—C16	123.1 (2)	N2—C16—C17	113.5 (2)
N1—C1—C2	131.0 (2)	N2—C16—H16A	108.9
N1—C1—C6	109.2 (2)	C17—C16—H16A	108.9
C2—C1—C6	119.8 (3)	N2—C16—H16B	108.9
C3—C2—C1	118.1 (3)	C17—C16—H16B	108.9
C3—C2—H2	121.0	H16A—C16—H16B	107.7
C1—C2—H2	121.0	C22—C17—C18	119.5 (2)
C2—C3—C4	121.5 (3)	C22—C17—C16	123.2 (2)
C2—C3—H3	119.2	C18—C17—C16	117.2 (2)
C4—C3—H3	119.2	O3—C18—C17	117.4 (2)
C5—C4—C3	121.3 (3)	O3—C18—C19	122.2 (2)
C5—C4—H4	119.4	C17—C18—C19	120.4 (2)
C3—C4—H4	119.4	O4—C19—C20	125.7 (2)
C4—C5—C6	116.9 (3)	O4—C19—C18	115.1 (2)
C4—C5—H5	121.6	C20—C19—C18	119.2 (2)
C6—C5—H5	121.6	C21—C20—C19	119.9 (3)
N2—C6—C5	131.3 (2)	C21—C20—H20	120.0
N2—C6—C1	106.2 (2)	C19—C20—H20	120.0
C5—C6—C1	122.4 (3)	C22—C21—C20	120.3 (3)
N1—C7—N2	111.9 (2)	C22—C21—H21	119.8
N1—C7—C8	121.6 (2)	C20—C21—H21	119.8
N2—C7—C8	126.5 (2)	C21—C22—C17	120.6 (2)
C9—C8—C13	118.6 (2)	C21—C22—H22	119.7
C9—C8—C7	117.8 (2)	C17—C22—H22	119.7
C13—C8—C7	123.4 (2)	O4—C23—C24	108.0 (2)
O1—C9—C8	122.7 (2)	O4—C23—H23A	110.1
O1—C9—C10	117.2 (2)	C24—C23—H23A	110.1
C8—C9—C10	120.1 (2)	O4—C23—H23B	110.1
O2—C10—C11	126.1 (2)	C24—C23—H23B	110.1
O2—C10—C9	114.0 (2)	H23A—C23—H23B	108.4
C11—C10—C9	119.9 (2)	C23—C24—H24A	109.5
C10—C11—C12	120.0 (3)	C23—C24—H24B	109.5
C10—C11—H11	120.0	H24A—C24—H24B	109.5
C12—C11—H11	120.0	C23—C24—H24C	109.5
C13—C12—C11	120.7 (3)	H24A—C24—H24C	109.5
C13—C12—H12	119.6	H24B—C24—H24C	109.5
C11—C12—H12	119.6	O5—N3—O6	123.0 (5)
C12—C13—C8	120.5 (3)	O5—N3—C25	121.8 (5)
C12—C13—H13	119.7	O6—N3—C25	115.2 (4)
C8—C13—H13	119.7	N3—C25—H25A	109.5
O2—C14—C15	107.1 (2)	N3—C25—H25B	109.5
O2—C14—H14A	110.3	H25A—C25—H25B	109.5
C15—C14—H14A	110.3	N3—C25—H25C	109.5
O2—C14—H14B	110.3	H25A—C25—H25C	109.5
C15—C14—H14B	110.3	H25B—C25—H25C	109.5
			
C7—N1—C1—C2	179.1 (3)	O1—C9—C10—O2	−3.3 (3)
C7—N1—C1—C6	−1.6 (3)	C8—C9—C10—O2	178.0 (2)
N1—C1—C2—C3	−179.9 (3)	O1—C9—C10—C11	175.6 (2)
C6—C1—C2—C3	0.8 (4)	C8—C9—C10—C11	−3.1 (4)
C1—C2—C3—C4	−0.1 (4)	O2—C10—C11—C12	179.3 (2)
C2—C3—C4—C5	−0.6 (5)	C9—C10—C11—C12	0.5 (4)
C3—C4—C5—C6	0.6 (4)	C10—C11—C12—C13	1.2 (4)
C7—N2—C6—C5	−178.3 (3)	C11—C12—C13—C8	−0.3 (4)
C16—N2—C6—C5	8.6 (4)	C9—C8—C13—C12	−2.2 (4)
C7—N2—C6—C1	1.0 (3)	C7—C8—C13—C12	−178.3 (3)
C16—N2—C6—C1	−172.1 (2)	C10—O2—C14—C15	−176.3 (2)
C4—C5—C6—N2	179.4 (3)	C7—N2—C16—C17	102.3 (3)
C4—C5—C6—C1	0.2 (4)	C6—N2—C16—C17	−86.3 (3)
N1—C1—C6—N2	0.3 (3)	N2—C16—C17—C22	−3.6 (4)
C2—C1—C6—N2	179.8 (2)	N2—C16—C17—C18	177.0 (2)
N1—C1—C6—C5	179.7 (3)	C22—C17—C18—O3	−179.6 (2)
C2—C1—C6—C5	−0.9 (4)	C16—C17—C18—O3	−0.2 (4)
C1—N1—C7—N2	2.3 (3)	C22—C17—C18—C19	−0.8 (4)
C1—N1—C7—C8	−178.9 (2)	C16—C17—C18—C19	178.6 (2)
C6—N2—C7—N1	−2.1 (3)	C23—O4—C19—C20	−12.0 (4)
C16—N2—C7—N1	170.3 (2)	C23—O4—C19—C18	168.1 (2)
C6—N2—C7—C8	179.1 (2)	O3—C18—C19—O4	−0.6 (4)
C16—N2—C7—C8	−8.4 (4)	C17—C18—C19—O4	−179.2 (2)
N1—C7—C8—C9	−25.1 (4)	O3—C18—C19—C20	179.5 (2)
N2—C7—C8—C9	153.6 (2)	C17—C18—C19—C20	0.8 (4)
N1—C7—C8—C13	151.1 (3)	O4—C19—C20—C21	179.5 (3)
N2—C7—C8—C13	−30.3 (4)	C18—C19—C20—C21	−0.6 (4)
C13—C8—C9—O1	−174.7 (2)	C19—C20—C21—C22	0.4 (4)
C7—C8—C9—O1	1.6 (4)	C20—C21—C22—C17	−0.4 (4)
C13—C8—C9—C10	3.9 (4)	C18—C17—C22—C21	0.6 (4)
C7—C8—C9—C10	−179.8 (2)	C16—C17—C22—C21	−178.7 (3)
C14—O2—C10—C11	−4.9 (4)	C19—O4—C23—C24	−172.3 (2)
C14—O2—C10—C9	173.9 (2)		

### Hydrogen-bond geometry (Å, º)

**Table:**

*D*—H···*A*	*D*—H	H···*A*	*D*···*A*	*D*—H···*A*
O1—H1*O*···N1	0.92 (4)	1.75 (4)	2.596 (3)	152 (3)
O3—H3*O*···O4	0.99 (3)	2.27 (3)	2.710 (2)	106 (2)
O3—H3*O*···O1^i^	0.99 (3)	1.91 (3)	2.819 (2)	151 (2)
O3—H3*O*···O2^i^	0.99 (3)	2.36 (3)	3.012 (3)	122 (2)

Symmetry code: (i) −*x*, *y*+1/2, −*z*+1/2.
